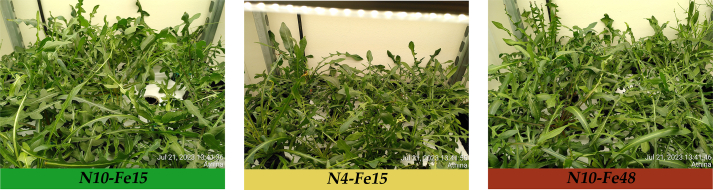# Optimizing vertical farm cultivation of *Cichorium spinosum* L.: White Light's influence and nutrition management

**DOI:** 10.1016/j.heliyon.2024.e37146

**Published:** 2024-08-30

**Authors:** Orfeas Voutsinos-Frantzis, Dimitrios Savvas, Georgios Liakopoulos, Ioannis Karavidas, Theodora Ntanasi, Leo Sabatino, Leo F.M. Marcelis, Georgia Ntatsi

**Affiliations:** a Laboratory of Vegetable Production, Department of Crop Science, Agricultural University of Athens, Iera Odos 75, 11855, Athens, Greece; b Laboratory of Plant Physiology, Agricultural University of Athens, Department of Crop Science, Iera Odos 75, 11855, Athens, Greece; c Department of Agricultural, Food and Forest Sciences (SAAF), University of Palermo, Viale delle Scienze, Ed. 5, 90128, Palermo, Italy; d Horticulture and Product Physiology Group, Wageningen University and Research, P.O. Box 16 6700AA, Wageningen, the Netherlands

**Keywords:** Stamnagathi, Vertical farming, Photosynthesis, White light, Leaf thickness, Stomata, Nutrient solutions, Iron, Nitrate, Nitrogen

## Abstract

The objective of this study was to examine the integration of a wild leafy vegetable, *Cichorium spinosum* L., in vertical farms. This research comprises two experiments focusing on different “white” light products and nutrient solutions. During both experiments, the temperature varied between 25 and 28 °C, relative humidity ranged from 50 to 70 %, carbon dioxide was at 450 ppm, and light intensity was set at 300 μmol m^−2^ s^−1^ respectively. In the lighting experiment, the three spectra used had the commercial names Neutral, Full and a SunLike™, and their spectral composition (blue:green:red:far-red) were 14:32:43:10, 16:36:40:8, and 21:34:36:7 respectively. The photoperiod was set to 12 h and the plant density was 50 plants m^−2^. Results showed no significant impact on agronomical parameters and leaf anatomy. The stomatal length and width decreased as the red:blue ratio of the light sources decreased, being greater in the Neutral treatment (red:blue ratio of 3.1) compared to the Full and SunLike™ (red:blue ratios of 2.5 and 1.7 respectively). Based on these results the preferable “white light” product was the one with the highest efficiency and lowest market price at the time of the experiment. In the nutrient solution experiment, the agronomical and nutritional attributes of stamnagathi plants supplied with a control nutrient solution, “N10-Fe15” were compared to plants cultivated under limited nitrogen, “N4-Fe15” and elevated iron, “N10-Fe48”, EC was 1.5 ds m^−1^, and pH was 5.6–6.5. The experiment simulated commercial practices by increasing the photoperiod to 15 h and plant density to 100 plants per square meter. The results did not demonstrate significant effect of the nutrient solution differences on the agronomical characteristics except from a decrease in total Kjeldahl nitrogen under limited nitrogen conditions. Notably, leaf tissue phosphorus content increased under elevated iron conditions. The nitrate content remained within safe for consumption thresholds for all treatments. Based on these results, stamnagathi can be integrated in vertical farms under limited nitrogen conditions. Stamnagathi's resilience to elevated iron in the nutrient solution demonstrated its potential for future biofortification experiments.

## 1 Introduction

“Vertical farming” is an umbrella term referring to the protected cultivation of plants in soilless culture systems with artificial lighting. These vertical farms can either have multiple horizontally stacked layers or vertical plant panels. From a commercial perspective, this technology allows for cultivation of a diversity of crops [[1], [2], [3], [4]]. Studies from the early 2000s revealed that red and blue LEDs could produce plant growth comparable to natural sunlight, owing to their effective photon absorption [5]. By 2010, advancements in phosphor technology enabled the development of white LEDs, which provide a broad spectrum light similar to that of natural sunlight [6,7]. This control over the light qualities, such as spectrum, photoperiod, and intensity opened up numerous opportunities towards regulating physiological properties in controlled environment agriculture [8,9]. Nonetheless, this development has created ample room for experimentation and has sparked a complex quest to find the optimal light characteristics for each plant, cultivar, and cultivation stage [10]. Furthermore, the extensive spectrum combinations explored in existing literature, including comparisons of R:B ratios ranging from 1 to 8 using monochromatic blue and red LEDs, combinations with green and white LEDs, or even fluorescent light [[11], [12], [13]], render it impossible to define one “optimum” spectrum. Nevertheless, LED efficiency remains a factor of high importance when it comes to choosing the right lighting for commercial production [[14], [15], [16]]. Regardless of the current advances of the lighting technology, the rapid growth of the industry has not been without its criticisms. High energy consumption and carbon emissions of vertical farms, in contrast to open field and greenhouse production [[17], [18], [19]], coupled with the challenges faced by leading companies in maintaining their growth and financial stability [20,21] have both been subjects of debate within scientific and industrial circles.

Despite these challenges, there is a growing trend towards integrating "niche" crops into vertical farming systems. These crops typically command higher prices in the market compared to mainstream crops [22,23]. In the modern world, the passion for improving the consumer's health has stirred the focus on "optimal nutrition" and "functional foods". This shift in consumer preferences sets the stage for the exploration of crops with potential health benefits. Throughout history, numerous plants have been highly valued for their abundance in mineral elements and bioactive compounds, making them frequent choices for consumption due to their potential health benefits [24,25]. While only a few plant-based foods have been sufficiently examined and meet scientific health standards, there's increasing evidence supporting the health advantages of various plant foods, including wild leafy greens [[26], [27], [28]]. Wild edible greens have historically been considered as “health promoting” [29], making them an interesting candidate for the “functional foods” category, especially for vertical farms. These wild edible greens offer a range of phytonutrients, bioactive compounds and potential anti-carcinogenic properties, and could play a key role in solidifying the success of certain vertical farming enterprises [[30], [31], [32]].

The natural origin of wild foraged plants does not always mean “safe” nor “healthy”. It is crucial to recognize potential risks such as toxic compounds and high heavy metal concentrations [[33], [34], [35], [36]]. By documenting the chemical profiles of these plants, the toxic compounds can be characterized and therefore plants with toxic attributes can be avoided. On the other hand, being grown spontaneously in the wild where none of the environmental parameters are controlled, nor monitored, the concentrations of certain elements and the health promoting compounds might also vary. Leafy vegetables are known to be the top nitrate-containing plant-based foods [37]. Towards that end, some wild leafy vegetables have often high nitrate content even when gathered from virgin lands [38]. Due to several concerns regarding the implications of nitrate intake in human health, such as gastric cancer [[39], [40], [41], [42]], nitrate accumulation in vegetables has been a topic of interest since the 70s [43,44]. At the same time, fruits and vegetables are considered beneficial towards cancer prevention [45,46]. More recent studies suggest that the nature of the dietary source (vegetable, meat, water) is important and that nitrates derived from vegetables can even be beneficial for cardiovascular health [47,48].

Certain thresholds have been established by the European Commission regarding nitrate levels in different leafy vegetables (regulation No 1258/2011) [49]. This precautionary measure derived from indications in epidemiological studies demonstrating a probable connection between nitrate intake from food sources and the appearance of a range of cancers [[50], [51], [52]]. Depending on the crop type and seasonality, the nitrate threshold can be as high as 5000 mg per kg of fresh vegetable weight. Preventing high nitrate levels in vegetables has been the focus of several studies when it comes to agricultural systems [53]. Given the degree of control over light quality and quantity in vertical farms, maintaining low nitrate levels in leaf tissues through the alteration of the spectral composition [54], light intensity [55] and photoperiod [56] has been found to be an effective way for the production of safe-for-consumption leafy greens. In addition, fertilization strategies, such as altering the total nitrogen concentration of the supplied nutrient solution [57], or replacing a percentage of the supplied nitrogen as nitrate with ammonium [58], have been found to be good agricultural practices that could help set the foundations for vegetables with low nitrate content.

This research focuses on the wild leafy edible plant *Cichorium spinosum* L., also referred as spiny chicory in literature, or as “stamnagathi” in Greek. Stamnagathi is rich in phytonutrients and secondary metabolites, indigenous to coastal regions near the Mediterranean Sea, including rocky coastal areas and mountainous terrain. It's leaves form a ground rosette and are usually handpicked depending on the growth stage and consumed boiled or sometimes raw in salads [[59], [60], [61], [62]]. Despite its richness in nutritional components, limited research has been conducted on this wild plant, with some studies focusing on open field production, while others focusing on its cultivation in soilless agricultural systems [[63], [64], [65], [66], [67]]. To our knowledge, this research marks the first exploration of stamnagathi's responses to light spectra and nutrition in the context of vertical farms.

In the scope that “white light” could be utilized to reduce the cost of the initial investment and allow more entrepreneurs to be a part of the vertical farming industry, “white light” LED fixtures, with distinct efficiency, spectral composition and initial costs were used.

In this research we investigated three spectra to assess their impact on the agronomical and morphological characteristics of stamnagathi, under a consistent light intensity. Our objective was to determine whether spectral differences alone would influence growth or morphology significantly enough to justify selecting one product over another based on its spectral qualities, rather than solely considering efficiency or initial price. Building on the initial study, a specific spectrum was chosen for a second experiment to assess: (a) if lowering nitrogen levels in the nutrient solution could reduce leaf nitrate content in stamnagathi plants without affecting yield, and (b) if higher iron levels in the solution could enhance growth and yield.

## 2 Results

### 2.1 Experiment 1: Assessing white light's influence on growth and leaf morphology of *C*. *spinosum* L. In a vertical farm

At the end of the lighting experiment, plants from all treatments had reached a stage of an average 18, 19, and 17 leaves plant ^−1^, covering a leaf area of 151, 173, and 166 cm^2^ plant^−1^ a fresh weight an average of 9.8, 10.1 and 10.2 g plant ^−1^ and having a dry weight of 1.0, 1.2 and 1.3 g plant^−1^ for S, N and F spectra respectively. Nevertheless, these differences were not statistically significant (Table 1, Fig. 1, Fig. 2 and Fig. S1). From the morphological characteristics’ measurements (Fig. 3), the average measured values were 472, 460, and 460 nm for the leaf thickness, 255, 253, and 234 μm for the palisade parenchyma thickness, 236, 230, and 225 μm for the spongy parenchyma thickness, for S, N, F spectra respectively. According to the statistical analysis, the observed values could not be attributed to the spectral differences (Table 2). The stomatal density for the S, N, and F spectra was 146, 159 and 150 stomata mm^−1^, respectively and did not differ significantly between plants cultivated in the three treatments. The values of the stomatal length and stomatal width were the only ones that were affected by the spectral qualities. As seen in Table 3, the stomatal length and stomatal width of the N treatment (20.0 and 9.13 μm) were greater compared to the F (25.9 and 12.9 μm), which in turn were greater compared to the S (21.9, and 10.1 μm).

**Table 1:** Effect of three different kinds of “white light”, a “SunLike” (S), a “neutral” (N), a “full” (F) and with B:G:R:FR ratios 21:34:36:7, 14:32:43:10 and 16:36:40:8, respectively, on leaf number (LN), leaf area (LA), leaf fresh weight (LFW), and leaf dry weight (LDW) of stamnagathi.

Treatment	LN (No plant⁻^1^)	LA (cm^2^ plant⁻^1^)	LFW (g plant⁻^1^)	LDW (g plant⁻^1^)
S	18	151	9.8	1.0
N	19	173	10.1	1.2
F	17	166	10.2	1.3
Statistical significance	ns	ns	ns	ns

For each parameter, values are means of 12 plants per replicate of each light treatment (*n* = 3). There were no statistically significant differences according to Duncan's multiple range test for p ≤ 0.05. No statistical significance difference is depicted with the “ns” in the last row of the table.

**Fig. 1:**
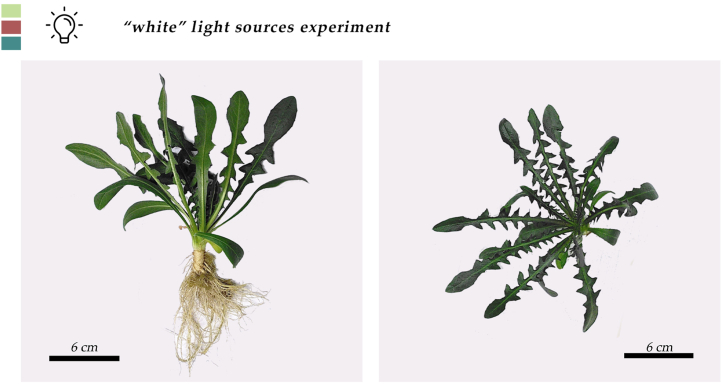
Side and top view of stamnagathi plant at the harvest stage cultivated under the SunLike™ spectrum. A ruler was used for scaling. No significant differences were observed between plants of different lighting treatments. Figure is indicative of stamnagathi's morphology.

**Fig. 2:**
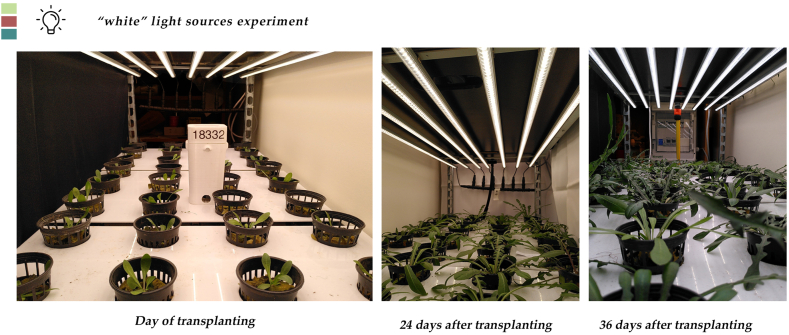
Growth stages of stamnagathi plants grown at a 50 plants m^−1^ density, from the transplanting day to harvest from the Neutral, Full and SunLike™ spectra respectively. Pictures are indicative. No visual differences between the “white” light spectra treatments were observed.

**Fig. 3:**
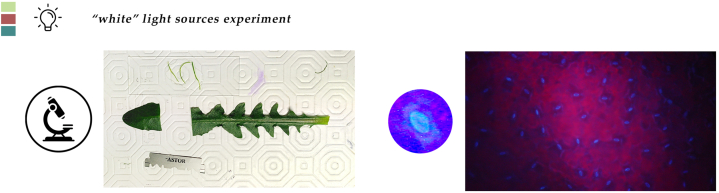
Leaf slices of the second youngest and fully expanded leaf for the microscopic observation of leaf total thickness, and palisade and spongy parenchyma thickness. Measurement of stomatal density stomatal length and width of the abaxial surface of fresh leaf specimens observed with incident UV light.

**Table 2:** Effect of three different kinds of “white light”, a “SunLike” (S), a “neutral” (N), a “full” (F) and with B:G:R:FR ratios 21:34:36:7, 14:32:43:10 and 16:36:40:8, respectively, on leaf thickness (LT), palisade parenchyma thickness (PT), and spongy parenchyma thickness (ST) of stamnagathi.

Treatment	LT (um)	PT (um)	ST (um)
S	472	255	236
N	460	253	230
F	460	234	225
Statistical Difference	ns	ns	ns

For each parameter, values are means of 6 plants per treatment cultivated under each light source (*n=*6). There were no statistically significant differences according to Duncan's multiple range test for p ≤ 0.05. No statistical significance difference is depicted with the “ns” in the last row of the table.

**Table 3:** Effect of three different kinds of “white light”, a “SunLike” (S), a “neutral” (N), a “full” (F) and with B:G:R:FR ratios 21:34:36:7, 14:32:43:10 and 16:36:40:8, respectively, on stomata density (SD), stomatal length (SL), and stomatal width (SW) of stamnagathi.

Treatment	SD (mm^−2^)	SL (um)	SW (um)
S	146	20.0 c	9.13 c
N	159	25.9 a	12.9 a
F	150	21.9 b	10.1 b
Statistical significance	ns	*	*

### 2.2 Experiment 2: Influence of limited nitrogen or elevated iron in nutrient solutions on *C*. *spinosum* L. growth and mineral content in a vertical

**Fig. 4:**
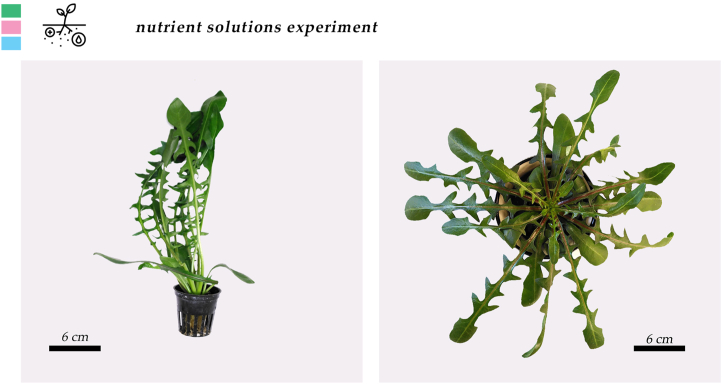
Side and top view of stamnagathi plant at the harvest stage cultivated under the control nutrient solution. For the top view photo, the plant was replaced into a bigger net pot. A ruler was used for scaling. No visual differences were observed between plants of different nutrient solution treatments. Figure is indicative of stamnagthi's morphology.

**Fig. 5:**
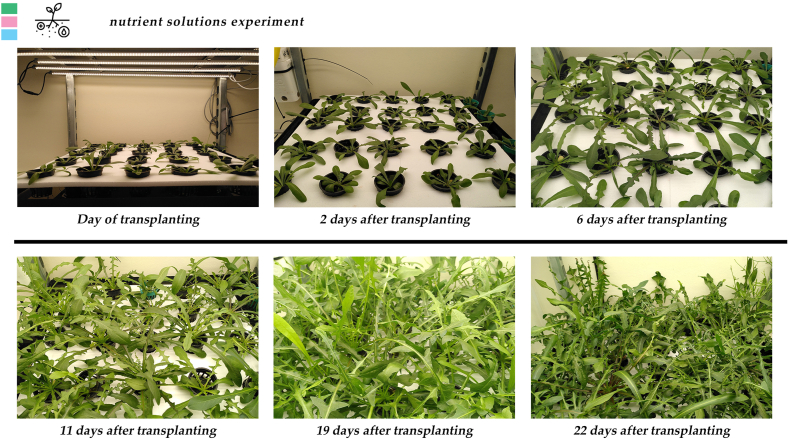
Growth stages of stamnagathi plants, grown at a 100 plants m⁻^1^ density, from the transplanting day to harvest. Pictures are indicative of the growth rate. No visual differences were observed between the different nutrient solution treatments.

Stamnagathi's agronomical characteristics were unaffected by the different nutrient solution treatments (Table 4, Fig. 4, Fig. 5, Fig. S2). The average observe leaf number was 20 leaves plant^−1^ for all treatments, the area was 347, 361, and 380 cm^2^ plant^−1^, average leaf fresh weight was 17.7, 18.9, and 20.6 g plant ^−1^ and dry weight was 1.3, 1.5, and 1.5 g plant^−1^, for N10-Fe15, N4-Fe15, N10-Fe48 respectively. According to the statistical analysis these differences could not be ascribed to the different composition of the nutrient solution.

**Table 4:** Effect of the different nutrient solutions (NS) on leaf number (LN), leaf area (LA), leaf fresh weight (LFW), and leaf dry weight (LDW) of stamnagathi. The NS treatments were designed based on their N and Fe content. The control (10 mmol L^−1^, and 15 μmol/L, N10-Fe15), the limited N (4 mmol L^−1^ and 15 μmol/L, N4-Fe15) and the elevated iron (10 mmol L^−1^ and 48 μmol/L, N10-Fe48).

Treatment	LN (No plant⁻^1^)	LA (cm^2^ plant⁻^1^)	LFW (g plant⁻^1^)	LDW (g plant⁻^1^)
N10-Fe15	20	347	17.7	1.3
N4-Fe15	20	361	18.9	1.5
N10-Fe48	20	380	20.6	1.5
Statistical significance	ns	ns	ns	ns

For each parameter, values are means of 12 harvested plants per replicate (*n* = 3)., cultivated with the different nutrient solution recipes. There were no statistically significant differences according to Duncan's multiple range test for p ≤ 0.05. No statistical significance difference is depicted with the “ns” in the last row of the table.

**Fig. 6:**
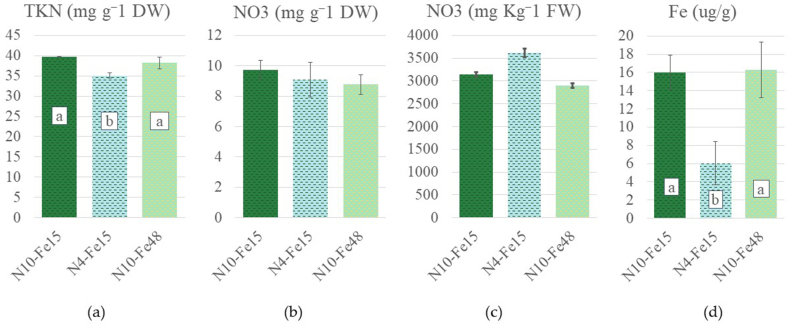
Chemical composition of average leaves of stamnagathi plants cultivated with three different nutrient solutions, N10-Fe15, N4-Fe15, and N10-Fe48. (a) Total Kjeldahl Nitrogen (TKN), expressed in mg g⁻^1^ of dry weight. (b) Nitrate content (NO₃) in dry (b) and fresh (c) leaves of stamnagathi expressed in mg g⁻^1^ of dry weight and mg Kg⁻^1^ of fresh weight respectively. (c) Iron (Fe) content expressed in μg g⁻^1^ of dry weight. Bars show means ± SE (*n* = 3). Different letters indicate statistically significant differences according to Duncan's multiple range test for p ≤ 0.05.

The chemical analysis of the total Kjeldahl N (Fig. 6a) demonstrated that compared to the 39.76 mg g^−1^ dry weight observed in the control, N10-Fe15, the values of N4-Fe15 were significantly decreased to 35.09 mg g^−1^ dry weight, whereas the total Kjeldahl N of N10-Fe48, 38.21 mg g^−1^ dry weight, did not differ significantly from either of the previous treatments. The NO₃ concentration in the leaf tissues (Fig. 6b and c) was not affected by neither the limited total-N nor the elevated Fe treatments.

**Fig. 7:**
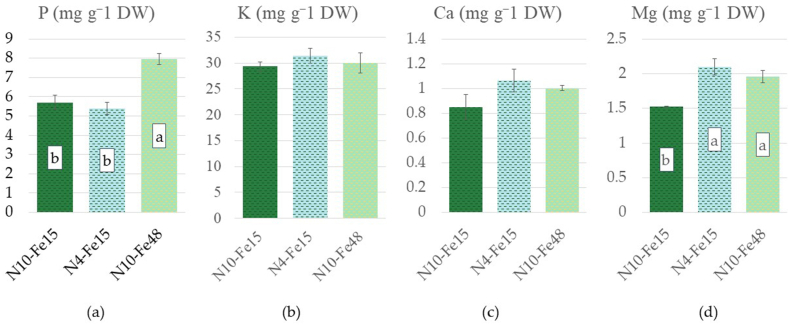
Chemical composition of average leaves of stamnagathi plants cultivated with three different nutrient solutions, N10-Fe15, N4-Fe15, and N10-Fe48. All values are expressed in mg g⁻^1^ of dry weight. (a) Phosphorus (P), (b) Potassium (K), (c) Calcium (Ca) and (d) Magnesium (Mg). Bars show means ± SE (*n* = 3). Different letters indicate statistically significant differences according to Duncan's multiple range test for p ≤ 0.05.

The leaf iron content was significantly affected by the nutrient solution treatments (Fig. 6d). In comparison to the control's observed value, 16.01 μg g^−1^ dry weight, the content of N4-Fe15 was reduced to 6.04 μg g^−1^ dry weight, whereas the content of the N10-Fe48, 16.26 μg g^−1^ dry weight, demonstrated a slight increase but not statistically significant to differentiate from the control. The phosphorus content was also significantly affected by the nutrient solution treatments. As observed in Fig. 7a, the P content of N10-Fe15 and N4-Fe 15 (5.69 and 5.39 mg g^−1^ dry weight) respectively, and was significantly surpassed by the content of N10-Fe48 which reached 7.94 mg g^−1^ dry weight. In addition, the K and Ca content (Fig. 7b and c) of the leaf tissues were 29 and 0.85, 31.33 and 1.06, 30 and 1.00 mg g^−1^ dry weight for N10-Fe15, N4-Fe15, N10-Fe48 respectively and were not significantly affected by the different nutrient solutions. On the contrary Mg levels (Fig. 7d) demonstrated an increase compared to the control (1.52 mg g^−1^ dry weight), in the leaf tissues of both the N4-Fe15 (2.09 mg g^−1^ dry weight) and N10-Fe48 (1.95 mg g^−1^ dry weight).

## 3 Discussion

### 3.1 Do spectral variations of different “white lights” justify product selection?

Light wavelengths of 450–495 nm (blue) and 620–750 nm (red) are often considered to be more efficient than wavelengths of 495–570 nm (green) in terms of quantum yield of CO_2_ assimilation [68]. A review on the importance of green light by Smith et al. [69], proposed that plants utilize these wavelengths not only to fine-tune stomatal aperture but also to refine overall canopy performance. Smith's results suggest that various functions of green light significantly enhance plant productivity and resource utilization, advocating for the incorporation of green wavelengths in LED-based agriculture, and therefore vertical farming. Several “white light” solutions currently exist on the market, often differentiating slightly in their spectral qualities. The efficiency of light capture and photosynthesis in a leaf is influenced by its structural components, their arrangement, and their intrinsic properties [70]. The absorption of photons varies with wavelength; green light, despite its lower absorption efficiency compared to red and blue light, can reach deeper into the leaves. This deeper penetration allows for more uniform chlorophyll excitation, potentially leading to higher yields, especially in high plant densities [71]. In a research by Zheng et al. [72], examining the impact of light wavelengths including red, blue, red and blue, and white light on three ornamental pot plants, observed that blue light significantly affects the total leaf thickness, though this observation might be species depended and the effects on the palisade and spongy parenchyma ratios may vary. In the same study, blue light increased stomatal density without affecting the stomatal aperture area. Stomatal size has been linked to the ratio of red and blue photons. It appears that the stomatal size decreases with the decrease of the R:B ratio as it has been observed in lettuce [73], cucumber seedlings [74], and soybean [75]. This observation is in agreement with our results, where the stomatal size was decrease as the R:B ratio of the N, F and S treatments was reduced from 3.1, to 2.5 and 1.7 respectively. In this study, leaf thickness, as well as the thickness of the palisade and spongy parenchyma, along with stomatal density, were observed to be unchanged by the light treatments. The observed leaf similarities, and differences resulted in non-significant differences on the overall agronomical characteristics. In our experiment, it was found that 36 days after transplant (DAT) and a total of 63 days after sowing (DAS), the FW of stamnagathi was around 10 g per plant, regardless of the light treatment. From an earlier greenhouse experiment conducted by our group, researchers reported yields of less than 6 g per plant after 56 days from transplanting (DFT) in a floating raft hydroponic system [65]. On the other hand, a more recent greenhouse experiment of our group had reported an increase in the yield leading to 9 g per plant after 21 DAT, and a total of 56 DAS [66]. In vertical farms it is important to utilize crops with small cultivation cycles or optimize the cultivation conditions to reduce the cultivation cycle [76]. The results of this experiment suggest that further optimization can be implemented for the reduction of the cultivation cycle of stamnagathi in vertical farms. Moreover, our results suggest that under “white light” the slight spectral differences did not demonstrate any significant agronomical effects that could justify the use of one product over the other. Hence the decision between the three, was based upon the efficiency and market price at the time (data not shown). Further investigation on the spectral quality combined with other environmental parameters, such as the effects of monochromatic LEDs, light intensity, and photoperiod, could be carried out in the future for the better understanding of the physiology of *C. spinosum* L.

### 3.1 Does limited nitrogen or elevated iron in the nutrient solution affect the agronomical characteristics of stamnagathi cultivated in a vertical farm?

Our results demonstrated that reducing the total nitrogen levels from 10.92 mmol L^−1^ (NO₃⁻: 9.28 mmol L^−1^ and NH₄⁺: 1.64 mmol L^−1^) of the control (N10-Fe15) to 4.55 mmol L^−1^ (NO₃⁻: 4.00 mmol L^−1^ and NH₄⁺: 0.55 mmol L^−1^) of the limited total-N treatment (N4-Fe15) did not have any significant effects on the agronomical characteristics of stamnagathi. In agreement to our results, in substrate based soilless culture of stamnagathi, Chatzigianni et al. [62], had observed that reducing the total-N from 16 mmol L^−1^ to 4 mmol L^−1^ in the nutrient solution of did not negatively affect the growth of stamnagathi plants. The leaf number and specific leaf area were not affected by the limited nitrogen, as it has been observed for lettuce [77]. The leaf fresh weight was similar for all treatments, suggesting that photosynthesis was maintained, either through prioritizing leaf expansion under limited nitrogen conditions [78] or stamnagathi, being a wild edible plant did not experience total nitrogen levels of 4.55 mmol L^−1^ as a significant limiting factor.

In a similar manner, the elevated Fe treatment (N10-Fe48) did not exhibit any discernible positive or negative effects. Filho et al. [79], while cultivating *Cichorium intybus* in an NFT soilless culture system, using different concentration of Fe (16 mmol L^−1^, 48 mmol L^−1^, 148 mmol L^−1^, and 448 mmol L^−1^) observed that the growth characteristics were negatively affected as the Fe concentrations increased but the optimal Fe concentration in the nutrient solution was identified in the range 48–148 mmol L^−1^. Further experimentation on Fe levels could define the optimal composition of the nutrient solution for stamnagathi cultivation, and enhance the database of the NUTRISENSE software. In addition, it has been observed by Hosseini et al. [80], that in vertical farming conditions the total fertilizer use, resulting in different EC levels, can be reduced in comparison to other soilless culture systems. This was also observed in the current research, where the EC level was decide to be less than that of previous experiments conducted by our group [58,66]. The current results, provide some initial details regarding the integration of stamnagathi in commercial vertical farming systems, suggesting that the nitrogen fertilizers could be reduced without affecting the overall growth, and elevating the iron levels does not result in yield losses. Future experiments could further focus on the EC levels and total fertilizer use per nutrient solutions recipe in order to define the parameters that could further enhance the agronomical characteristics, and primarily fresh weight per plant.

### 3.2 Does limited nitrogen or elevated iron in the nutrient solution affect the nutrient composition of stamnagathi leaves cultivated in a vertical farm?

Findings on the total leaf nitrogen content in the presented study were in agreement with the experimental hypothesis. The plants from the limited nitrogen treatment (N4-Fe15) had significantly reduced total nitrogen values to the control nitrogen treatments (N10-Fe15 and N10-Fe48). The observed values and leaf nitrogen content were consistent with the findings of Chatzigiani et al. [58] where 4.00 mmol L^−1^ of total-N in the supplied nutrient solution in perlite bags led to a decrease in the total Kjeldahl nitrogen concentration in stamnagathi leaves, compared to 16.00 mmol L^−1^. In contrast to our findings regarding the nitrate levels, where the different total nitrogen level of the nutrient solution did not affect the leaf nitrate content, Chatzigianni et al. [58], found that 4.00 mmol L^−1^ of total-N in the supplied nutrient solution in perlite bags decreased the nitrate concentration in stamnagathi leaves compared to those supplied with 16.00 mmol L^−1^ of total-N. In our case, this could perhaps be ascribed to the cultivation system. In vertical farms, the stability of the light intensity and photoperiod can maximize nitrate assimilation capacity of plants [81]. It is highly probable that the resulting unaffected nitrate content was primarily determined by the light conditions of the vertical farming lighting system rather than the nutrient solution characteristics [82]. Moreover, leaf nitrate content of stamnagathi plants in our experiment was below the suggested threshold of 4000 mg of nitrate per kg of fresh weight, and therefore were safe for consumption. Even though the leaf nitrate content calculated in the leaf fresh weight did not demonstrate statistically significant differences, values ranged from 2897, 3145 and 3618 mg of nitrate per kg of fresh weight for the N10-Fe15, N4Fe15 and N10-Fe48 respectively.

Additionally, the elevated Fe in the supplied nutrient solution (48 μmol/L) did not affect the total Kjeldahl nitrogen levels of the N10-Fe48 treatment, suggesting that this observed increase was solely ascribed to total-N. An increase of the leaf Fe content was observed in the N10-Fe15 and N10-Fe48 treatments compared to the N4-Fe15. This observation suggests the importance of total-N rather than the Fe concentration in the nutrient solution. In nitrate assimilation, Fe assumes a pivotal role by acting as a cofactor for enzymes within the reductive assimilatory pathway. These enzymes include nitrate reductase (NR), nitrite reductase (NiR), and glutamate synthase (GOGAT), all of which require Fe in the form of either an Fe-heme group or an Fe-S cluster [[83], [84], [85], [86], [87]]. In this context, it is believed that the elevated Fe observed in the treatments supplied with high total-N (10 mmol L^−1^) could be ascribed to the plant's need to assimilate the excess nitrate. This increase in assimilated nitrate is also evident in the total Kjeldahl nitrogen levels, which were higher in the treatments supplied with high levels of total-N and might be further reinforced by the insignificant differences in nitrate levels among the leaf tissues of the three treatments. It has been suggested that elevated Fe could increase the leaf N, P, K, Ca, and Mg contents of lettuce [88,89]. Nevertheless, in this research, only the P content of the leaves was increased solely due to elevated iron conditions. Towards that end, P content of stamnagathi plants cultivated under N10-Fe48 was significantly higher compared to N10-Fe15 and N4-Fe15, suggesting that higher Fe levels in the supplied nutrient were accompanied by higher leaf P content. Even though, K, Ca demonstrated insignificant differences between plants of the different treatments, the Mg content appeared lower in plants of the N10-Fe15 treatment.

These results demonstrate that limited nitrogen on the chemical composition of stamnagathi primarily affects total nitrogen and iron concentration. Future investigation on the interaction of nutrient solution composition and light intensity could lead to cultivation protocols for stamnagathi plants with lower nitrate content. Increasing the iron content of the nutrient solution primarily affected the phosphorus content of the leaf tissues. Increasing the leaf content of essential micronutrient, such as iron, has been proposed as a means to combat hidden hunger. Given the growing interest for vegetables with fortified mineral and bioactive content, stamnagathi could be utilized as a wild edible plant with low nitrogen requirements and resilience towards iron concentration in the nutrient solution. Further investigation on elevated iron concentrations in the nutrient solution (up to 2.0 mM Fe) could validate stamnagathi as a potential biofortified product.

## 4 Conclusions

Different lighting products have been presented in the vertical faring industry resulting in chaos and confusion for growers and intrapreneurs. White light consists of photons throughout the visible spectrum and can be applied to range of different plants. In the first experiment, three different “white light” products were tested on the cultivation of stamnagathi in a vertical farm. The spectral differences did not appear to affect the agronomical characteristics of stamnagathi. The average plant weighted 10 g, and at a plant density of 50 plants m^−2^ the yield (Kg m^−2^) was estimated to be around 0.5 kg m^−2^ regardless of the light source. The leaf morphology remained mostly unaffected as leaf and mesophyll thickness, and stomatal density did not differ significantly between the three light sources. The dimensions of the stomatal pores, were the only parameters of the lighting experiment that demonstrated significant differences. The stomatal length and width were found to be positively affected by the red:blue ratio. As the red:blue ratio increased from 1.7, in the SunLike™, to 2.5, in the Full, and 3.1, in the Neutral treatments. Based on these results, the decisive factor towards the selection of the most suitable “white light” product was efficiency market price at the time of the current study.

The challenge of lowering fertilizer usage without impacting yields has been a key area of study over the past few decades. Moreover, the possibility of biofortifying plants with micronutrients, such as iron, has been suggested as a method to combat hidden hunger. Towards that end, the second experiment explored the effects of total nitrogen reduction (N4-Fe15) and increased iron concentration (N10-Fe48) in the supplied nutrient solution compared to a control solution (N10-Fe15). Additionally, increasing the plant density and photoperiod based on previous research, the second experiment aimed to further increase the overall yield of stamnagathi production in vertical farms beyond research conditions. The agronomical traits of stamnagathi were unaffected by differences in nutrient solution composition, demonstrating its resilience to nitrogen limitation and its tolerance to elevated iron levels. The average plant weighted 18 g, at a plant density of 100 plants m^−2^, resulting in a total yield of 1.8 kg m^−2^ regardless of the supplied nutrient solution. Moreover, the chemical analysis of the leaf plant tissues indicated a decrease in total Kjeldahl nitrogen under limited nitrogen conditions (N4-Fe15), while elevated iron (N10-Fe48) did not show a significant impact. Notably, leaf tissue phosphorus was positively associated with elevated iron conditions (N10-Fe48) in the nutrient solution. The nitrate content remained within safe for consumption thresholds for all treatments. Our results showcase stamnagathi as a prime candidate for further experimentation in vertical farms, towards fertilizer use reduction and iron biofortification.

## 5 Materials and methods

### 5.1 Plant material and seedling preparation

As plant material, stamnagathi (*Cichorium spinosum* L.) seeds of the mountainous ecotype were used. For both experiments the sowing took place on AO 25/40 rockwool plugs (Grodan, Roermond, Τhe Netherlands). Each plug was 25*25*40 mm and connected to the others forming a sheet of 200 plugs. During the seedling preparation stage these rockwool sheets were placed in the ebb and flow trays of the Vegeled trolley (Vegeled™ by Collasse SA, Seraing, Belgium). Temperature, relative humidity, and carbon dioxide concentration were measured by a Sigrow Pro sensor (Sigrow B.V., Wageningen Campus, Wageningen, The Netherlands). The average temperature, relative humidity and CO_2_ values were 25 °C, 60 %, and 400 ppm during the light period and 18 °C, 50 %, and 360 ppm during the dark period. For the seedling preparation stage the photoperiod was 12 h. For the lighting experiment, the seedlings were prepared under the light spectrum that they would continue to grow after separation and transplanting of the rockwool plugs. Hence, the treatments effect had started from sowing. For the nutrient solution experiment, the seedlings were prepared only under the “Neutral” spectrum with the following Blue:Green:Red:Far-red compostion 14:32:43:10. Seedlings for both experiment were prepared under a light intensity of 180 μmol m^−2^ s^−1^.

The rockwool sheets were irrigated once at the beginning of the germination phase and then sprayed every 2–3 days to maintain moisture. Almost a week after sowing, as the seeds started to sprout and the cotyledons were visible, fertigation with the control nutrient solution was automatically applied through the ebb and flow system of the Vegeled trolley. The composition of the supplied nutrient solution was the control (N10-Fe15, Table 6). At the same time, seedlings were removed with tweezers form plugs that had more than one sprouted seed. For the next 10 days, fertigation was applied for 2 min every 2 days. After that, and for another 10 days, fertigation frequency was increased to 2 min per hour. Separation and transplanting of the seedlings were conducted 27 days after sowing, when the plants had reached the stage of 3–7 true leaves. Individual plugs were inserted into plastic net pots and distributed to each treatment of each experiment. During transplanting, seedlings were sorted by leaf count, ensuring that those selected for the experiments had between 4 and 6 true leaves.

**Table 5:** Spectral composition of the three different “white lights”.

Color	Neutral	Full Spectrum	Sunlike
Blue %	14	16	21
Green %	32	36	34
Red %	43	40	36
Far Red %	10	8	7
R:B	3.1	2.5	1.7
G:B	2.3	2.2	1.6
R:FR	4.3	5	5.1
Efficiency μmol J^−1^	2.4	2.4	1.8

**Table 6:** Chemical composition of the nutrient solution of each treatment.

Nutrient solution parameter	Units	N10-Fe15	N4-Fe15	N10-Fe48
EC	ds m⁻^1^	1.49	1.48	1.49
pH		5.6	5.6	5.6
K⁺	mmol L⁻^1^	4.23	4.81	4.23
Ca^2^⁺	mmol L⁻^1^	2.32	2.48	2.32
Mg^2^⁺	mmol L⁻^1^	1.01	1.09	1.01
NH₄⁺	mmol L⁻^1^	1.64	0.55	1.64
NO₃⁻	mmol L⁻^1^	9.28	4	9.28
H₂PO₄⁻	mmol L⁻^1^	1.06	1.06	1.06
SO₄^2^⁻	mmol L⁻^1^	0.70	2.85	0.70
Fe	μmol L⁻^1^	15.36	15.36	48
Mn⁺⁺	μmol L⁻^1^	8.64	8.64	8.64
Zn⁺⁺	μmol L⁻^1^	3.84	3.84	3.84
Cu⁺⁺	μmol L⁻^1^	0.67	0.67	0.67
B	μmol L⁻^1^	24	24	24
Mo	μmol L⁻^1^	0.48	0.48	0.48
Si	mmol L⁻^1^	0	0	0
Cl⁻	mmol L⁻^1^	1.92	1.92	1.92
Na⁺	mmol L⁻^1^	0.6	0.6	0.6
HCO₃⁻	mmol L⁻^1^	0.4	0.4	0.4

### 5.2 Experimental design of the lighting experiment

**Fig. 8:**
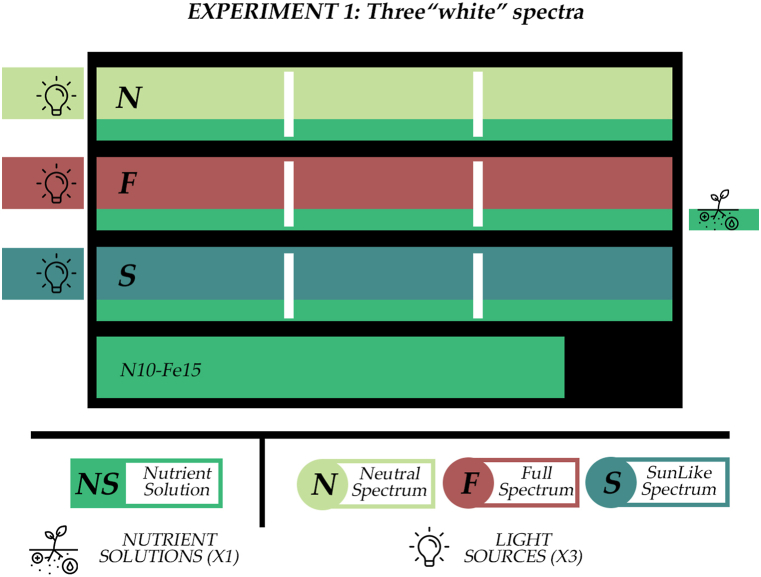
Illustrative depiction of the lighting experiment. In this experiment each layer had a different spectrum of “white light”, a “neutral” (N), a “full” (F) and a “SunLike” (S) but all layers were supplied from the same tank, with the same nutrient solution (NS). Each treatment had 3 replicates per layer. No randomization was implemented in this experiment.

**Fig. 9:**
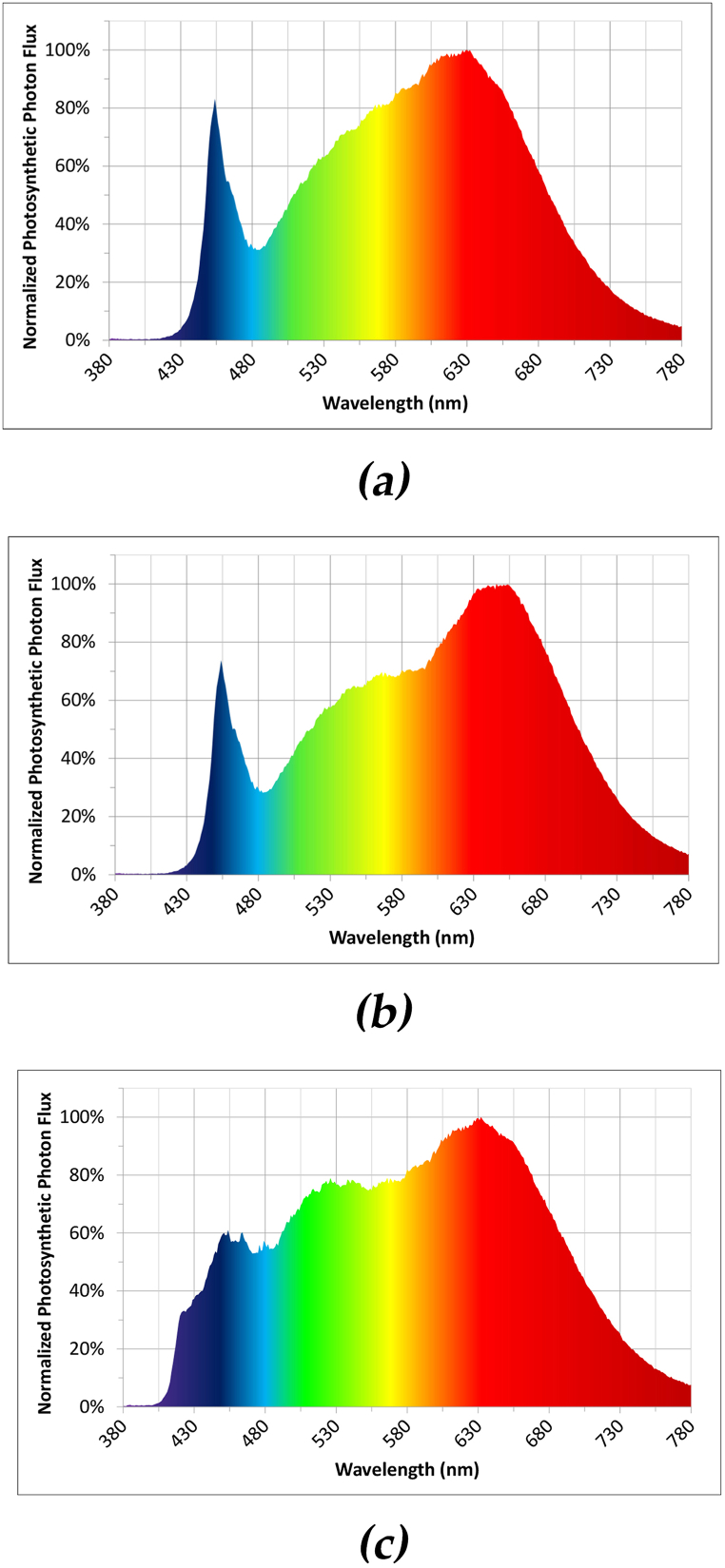
Normalized Photosynthetic Photon Flux for spectra (a) Neutral, (b) Full spectrum, and (c) Sunlike provided by Colasse.

The lighting experiment was conducted on a 135 × 56 cm Vegeled™ trolley with three layers and one common tank with nutrient solution (Fig. 8). The seedlings were transplanted into plastic net pots at a density of 50 plants m^−2^. Each layer was divided into three sections measuring 45 × 56 cm, with each section considered as one replicate. The lighting systems were Vegeled™ Eos series and had the commercial names “Neutral” (N), “Full” (F) and “SunLike™” (S). The light composition (Blue:Green:Red:Far-red) of the three spectral treatments was 14:32:43:10, 16:36:40:8, and 21:34:36:7 for the N, F and S treatments respectively (Table 5, Fig. 9). The efficacy of the LEDs was 2.4 μmol/J for N and F, and 1.8 μmol/J for S. Each layer had only one spectrum and there was no randomization in the lighting conditions. During the cultivation phase the climatic conditions were measured on each layer by an Air-Pro Sigrow sensor. During the light period, the average temperature was 27 °C, dropping to 25 °C during the dark period, with relative humidity at 68 % and 55 %, and CO_2_ levels at 450 ppm and 360 ppm, respectively. The photoperiod was 12 h long and the light intensity 300 μmol m^−2^ s^−1^. A minor temperature difference of about 1 °C, between the bottom and the top layer. The irrigation method used was, as in the seedling stage, “ebb and flow” and the nutrient solution was supplied to all layers simultaneously from one tank, drained back to the tank and recirculated. The chemical composition of the nutrient solution was consistent with that used in the seedling stage (N10-Fe15). The irrigation frequency was 5 min every half hour. The first irrigation was at 09:00–09:05 and the last at 21:00–21:05, at the end of the photoperiod. During each irrigation cycle the water level reached the bottom half of the rockwool plugs.

### 5.3 Experimental design of the nutrient solution experiment

**Fig. 10:**
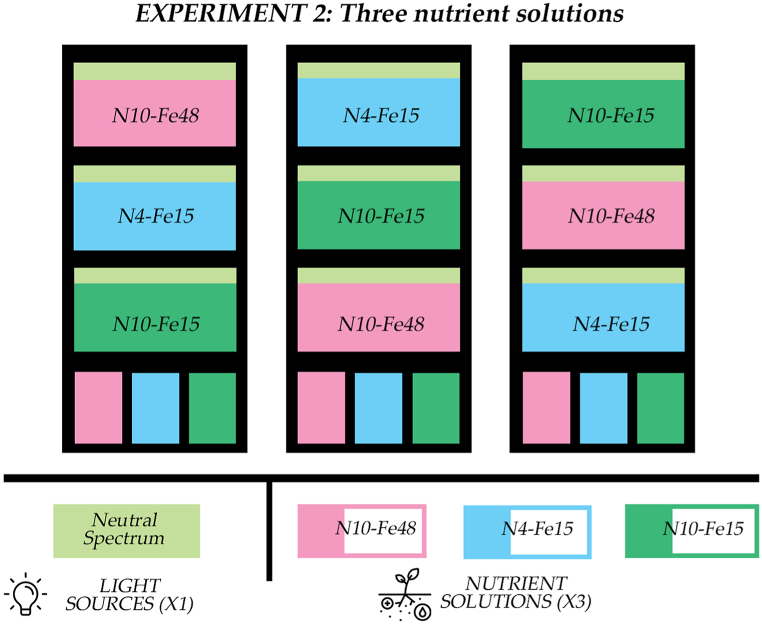
Illustrative depiction of the nutrient solution experiment. In this experiment the N spectrum was used for all layers and all cultivation units. Three NS treatments were carried out, based on their total-N and Fe content. The control (10 mmol L⁻^1^ and 15 μmol L⁻^1^, N10-Fe15), the limited total-N (4.55 mmol L⁻^1^ and 15 μmol L⁻^1^, N4-Fe15) and the elevated Fe (10 mmol L⁻^1^ and 48 μmol L⁻^1^, N10-Fe48). Each treatment had 3 replicates placed randomly in the three cultivation units.

The second experiment explored solely the nutrition aspect of the cultivation and was carried out on three 71.5 × 56.5 cm Vegeled™ trolleys. Each trolley had three layers and each layer was connected with one nutrient solution tank independently from the rest (Fig. 10). In the scope of increasing the productivity per square meter, the seedlings were transplanted into plastic net pots at a density of 100 plants m^−2^. The nutrient solution was supplied to each layer by “ebb and flow”. All nutrient recipes used in this experiment were prepared using the online software “NUTRISENSE DSS” (www.nutrisense.online, [90]) to have the same EC level (1.5 dS m⁻^1^). The three treatments were named after their total nitrogen and iron content and were as follows: the control treatment, N10-Fe15, with a total nitrogen content of 10 mmol L⁻^1^ and an Fe content of 15 μmol L⁻^1^, the limited total nitrogen treatment (N4-Fe15) which had a N content of 4 mmol L⁻^1^ and Fe 15 μmol L⁻^1^, and the elevated iron treatment (N10-Fe48), which had a total nitrogen content of 10 mmol L⁻^1^ and a Fe content of 48 μmol L⁻^1^ (Table 6). In all treatments, Fe was supplied as Fe-EDDHA (6 % Fe). The treatments were randomly positioned in the three trolleys following a randomized design. The irrigation frequency was increased to 20 min every hour. The first irrigation was at 09:00–09:30 and the last at 00:00–00:30, at the end of the photoperiod. The water level of each irrigation cycle was the same as in the lighting experiment but the tray cover was a 1 cm higher, holding the net pots a 1 cm higher. Hence, in the nutrient solution experiment the water level reached the bottom of the rockwool plugs and there was a 1 cm space between the end of the pot and the surface of the “ebb and flow” tray. After the results regarding the effect of light spectrum of the three products on stamnagathis’ growth, the lighting fixtures used in the nutrient solution experiment were Vegeled™ Eos series solely of the “Neutral” spectrum with the Blue:Green:Red:Far-red composition 14:32:43:10 and 2.4 μmol J⁻^1^ efficacy. The photoperiod was set to 15 h, after results of a parallel research demonstrated that stamnagathi could be cultivated under long photoperiods without flowering before reaching the commercial stage [91]. The light intensity was 300 μmol m^−2^ s^−1^. The environmental parameters averaged 28 °C and 25 °C for temperature, 70 % and 60 % for relative humidity, and 450 ppm and 360 ppm for carbon dioxide concentration during the light and dark periods, respectively. Table 7 provides a summary of the conditions for the seedling stage, lighting, and nutrient solution experiments.

**Table 7:** Synopsis of average environmental conditions during the light and dark periods (day and night) of the two experiments.

**Parameter**	Seedling stage	Lighting Experiment	Nutrient Solution Experiment
Temperature °C (Day/Night)	25/18	27/25	28/25
Humidity % (Day/Night)	60/50	68/55	70/60
CO_2_ ppm (Day/Night)	400/360	450/360	450/360
Light Intensity μmol m^−2^ s^−1^	180	300	300
Irrigation method	Top spray, then Ebb and flow	Ebb and flow	Ebb and flow
Photoperiod (hours)	12	12	15
Irrigation frequency	Depends on stage	5 min 0.5 h^−1^	30 min hour^−1^
Plant density (plants m^−2^)	1100	50	10
Spectra	Depends on experiment	N, F, S	N
Nutrient solutions	N10-Fe15	N10-Fe15	N10-Fe15, N4-Fe15 and N10-Fe48

The spectra refer to commercial names “Neutral” (N), “Full” (F) and “SunLike™” (S). The spectral composition (Blue:Green:Red:Far-red) of the three light treatments was 14:32:43:10, 16:36:40:8, and 21:34:36:7 respectively. The nutrient solution names refer to the concentrations of total nitrogen and iron in the nutrient solutions, N10-Fe15 (10 mmol L^⁻1^ and 15 μmol L^⁻1^), N4-Fe15 (4 mmol L⁻1, and 15 μmol L^⁻1^), and N10-Fe48 (10 mmol L^⁻1^ and 48 μmol L^⁻1^).

### 5.4 Plant measurements: agronomical, and morhological characteristics

During the cultivation period of both experiments, the nutrient solution volume, EC and pH were measured diurnal. For the measurement of the volume a ruler was used whereas Bluelab pens (Bluelab, Tauranga, New Zealand) were used to check the electrical conductivity and pH. Adjustments were made when values deviated from the target range (EC 1.5–2 ds/m and pH 5.5–5.6), and additional nutrient solution was added to the tank when the water level dropped significantly, potentially compromising the pump's ability to uniformly supply the solution. The time of harvest was decided based on two pillars. The first one was that the plants need to reach the minimum marketable size, the other was that, for an economically feasible production of stamnagathi, the total cultivation period should not exceed by far a two-month period. In the lighting experiment the plants were harvested 36 days after transplant. In the nutrient solution experiment the plants were harvested 24 days after transplant (Table 8). At harvest, the leaf quantitative characteristics (number, area and fresh weight) were measured, by separating leaves by hand and counting them, pacing them on the belt of the LI-3100C (LI-COR, Inc. Lincoln, NE, USA) and then measuriung their weight on a Mettler PE-3600 scale (Mettler Toledo LLC, Columbus, Ohio, USA). For the light experiment, 8 plants per layer, per replicate were harvested (resulting in 24 plants per treatment) whereas for the nutrient solution experiment 12 plants per layer, per replicate (resulting in 36 plants per treatment). For the drying process, individual paper bags were placed for seven days at 65 °C in STF-N 400 oven (FALC Instruments S.L.R, Treviglio, Italia). The dry weight was recorded after measuring the same dry weight for two consecutive days, indicating that the drying period ended and there was no more water left in the leaf tissues. Each replicate's samples were combined and finely ground by pulverizing them using the MF 10 Microfine grinder at its highest speed setting of 6000–6500 rpm (IKA Werke, Staufen, Germany). The grated leaf tissues were meticulously gathered into sealable plastic bags to protect the samples from moisture-related degradation. In addition, measurements of leaf characteristics such as leaf thickness, spongy and palisade parenchyma thickness, stomata density and stomata size were performed in 2 plants per replicate. The second youngest and fully expanded leaf was chosen for this measurement. For measuring leaf thickness, fresh transverse sections from the middle of the lamina were examined under a Zeiss Axiolab microscope (Carl Zeiss, Jena, Germany), with the thickness of the palisade and spongy parenchyma measured using a calibrated eyepiece. Stomatal density, length, and width were measured on the abaxial surface of 6 fresh leaf samples under incident UV light (maximum energy of 365 nm). To enhance the accuracy of stomatal pore measurements, 6 images per sample were captured (3 at 10x and 3 at 40x magnification) using a SSCD 38P/45 digital camera (SONY Corporation, Japan) and stored in digital format. The images were then edited in Image Pro-Plus to determine the number (at the 10x magnification) and the length of the stomata (40x magnification). In both experiments the remaining plants were collected, and their total fresh weight was assessed to refine the overall yield estimate.

**Table 8:** Timeline of each experiment and experimental phase.

**Action**	Experiment 1	Experiment 2
Sowing	December 23, 2022	June 02, 2023
Transplanting	January 19, 2023 (27 DAS)	June 29, 2023 (27 DAS)
Harvest	February 24, 2023 (36 DAT)	July 22, 2023 (24 DAT)
Total days	63 (DAS)	51 (DAS)

DAS stand for “days after sowing” and DAT for “days after transplanting”.

### 5.5 Chemical composition of leaf tissues and nutrient solutions

In the second experiment the chemical composition of the leaf tissues at the harvest stage was determined. For the determination of the leaf phosphorus (P), calcium (Ca), magnesium (Mg), iron (Fe), potassium (K), and sodium (Na) content, the grated leaf tissues for each replicate were processed with the dry ashing method. After grating the dried samples of each replicate of each treatment, subsamples of 0.5 g were placed in porcelain cups and subjected to 550 °C for 8 h in a chamber furnace LM-112 (Linn High Therm, Hirschbach, Germany). The ash was then dissolved in 1N HCL solution. The cup content was filtered through 125 mm Macherey-Nagel filter papers and transferred to 100 ml volumetric flasks that were then filled up to 100 ml resulting in the final aqueous leaf extracts which were transferred to 100 ml plastic bottles to be stored in a refrigerator at 2–4 °C. The chemical analysis were conducted in a three-day period after the extraction was carried out. Phosphorus levels were analyzed using the molybdenum blue colorimetric reaction, with absorbance recorded at 880 nm using an Anthos Zenyth 200 spectrophotometer (Biochrom Ltd, Cambridge, UK) [92,93]. The concentrations of Ca, Mg and Fe were determined through Flame Atomic Absorption Spectroscopy (FASS) [94] using the Atomic Absorption Spectrometer Shimadzu AA-7000 (Shimadzu, Kyoto, Japan) and carrying out dilutions of the samples, so that the values were withing each measurement's calibration curve. The K and P levels were determined through flame photometry [95], by preparing the calibration curve for K and Na, and conducting the required dilutions for the samples before placing them in the Sherwood Flame Photometer 410 (Sherwood, Cambridge, UK).

The nitrate (NO₃⁻) content in dry leaf tissues, was determined through the nitration of salicylic acid method [96]. For the extraction, two technical replicates were conducted by transferring two subsamples of 0.1 g, per sample, placing them in 15 ml centrifuge tubes and adding 10 ml of distilled water. Tubes were immersed in a 45 °C water bath for 1 h, stirring every 15 min. After filtering through 125 mm Macherey-Nagel filters, the solution was placed in sterilized 15 ml tubes. Following this, 0.2 ml of the solution was transferred to 25 ml centrifuge tubes, with 0.8 ml of salicylic and sulfuric acid solution added. The mixture was stirred for 20 min using an orbital shaker Unitwist 300 (Biotech, Madrid, Spain). After adding 19 ml of 2N NaOH solution, the samples were stirred for an additional 20 min. Identical treatments were applied to the standard samples for calibration. All samples and standards were then analyzed at 410 nm using the specified spectrophotometer. For the determination of nitrate in the fresh leaf tissues, the NO₃⁻ content of the dry leaf tissues was converted by multiplying with the dry weight/fresh weight ratio. The total Kjeldahl nitrogen was determined through digestion and distillation and manual titration. This process was carried out using the Labtec DT 220 apparatus in conjunction with the Scrubber Labtec SR 210 and Tecator Kjeltec 8200 equipment ([97], FOSS Analytical A/S, Hillerød, Denmark). Following distillation, each sample was manually titrated by measuring the volume (ml) of 0.05 N HCl solution required to change the solution's color from green to pink, thereby completing the determination of total Kjeldahl nitrogen.

### 5.6 Statistical analysis

Both the lighting experiment, which was not randomized, and the nutrient solution experiment, which was randomized, underwent One-Way ANOVA analysis using the Statistica 12 software package for Windows (StatSoft Inc., Tulsa, OK, USA). Duncan's multiple range test was administered at a significance level of p ≤ 0.05 for all measured variables.

## Data availability statement

The data are included in this paper.

## CRediT authorship contribution statement

**Orfeas Voutsinos-Frantzis:** Writing – review & editing, Writing – original draft, Visualization, Validation, Software, Methodology, Investigation, Formal analysis. **Dimitrios Savvas:** Writing – review & editing, Validation, Resources, Project administration, Methodology, Funding acquisition. **Georgios Liakopoulos:** Writing – review & editing, Validation, Methodology. **Ioannis Karavidas:** Writing – review & editing, Validation, Formal analysis, Data curation. **Theodora Ntanasi:** Writing – review & editing, Visualization, Software, Formal analysis. **Leo Sabatino:** Writing – review & editing, Validation, Methodology. **Leo F.M. Marcelis:** Writing – review & editing, Methodology, Data curation. **Georgia Ntatsi:** Writing – review & editing, Writing – original draft, Validation, Supervision, Software, Resources, Project administration, Methodology, Investigation, Funding acquisition, Data curation.

## Declaration of competing interest

The authors declare the following financial interests/personal relationships which may be considered as potential competing interests: Georgia Ntatsi, the coresponding author of this manuscipt, is Associate Editor for Heliyon Agriculture (a section of Heliyon). If there are other authors, they declare that they have no known competing financial interests or personal relationships that could have appeared to influence the work reported in this paper.

## Figures and Tables

**figs1:**
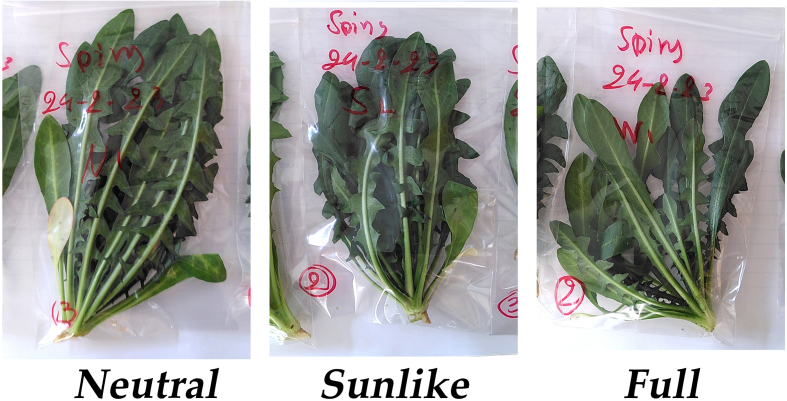


**figs2:**